# The effect of video-instructed versus audio-instructed dispatcher-assisted cardiopulmonary resuscitation on patient outcomes following out of hospital cardiac arrest in Seoul

**DOI:** 10.1038/s41598-021-95077-5

**Published:** 2021-07-30

**Authors:** Hee Soon Lee, Kicheol You, Jin Pyeong Jeon, Chulho Kim, Sungeun Kim

**Affiliations:** 1 EMS Situation Management Center, Seoul Emergency Operation Center, Seoul Metropolitan Fire & Disaster Headquarters, Seoul, South Korea; 2 Department of Emergency Medicine, Hallym University College of Medicine, Chuncheon, South Korea; 3 Department of Neurosurgery, Hallym University College of Medicine, Chuncheon, South Korea; 4 Department of Neurology, Hallym University College of Medicine, Chuncheon, South Korea; 5 Department of Emergency Medicine, EMS Situation Management Center, Seoul Emergency Operation Center, Seoul Metropolitan Fire & Disaster Headquarters, Toegye-ro 26-gagil 6, Jung-Gu, Seoul, Republic of Korea

**Keywords:** Cardiology, Health care, Medical research, Risk factors

## Abstract

We aimed to investigate whether video-instructed dispatcher-assisted (DA)-cardiopulmonary resuscitation (CPR) improved neurologic recovery and survival to discharge compared to audio-instructed DA-CPR in adult out-of-hospital cardiac arrest (OHCA) patients in a metropolitan city with sufficient experience and facilities. A retrospective cohort study was conducted for adult bystander-witnessed OHCA patients administered DA-CPR due to presumed cardiac etiology between January 1, 2018 and October 31, 2019 in Seoul, Korea. The primary and secondary outcomes were the differences in favorable neurologic outcome and survival to discharge rates in adult OHCA patients in the two instruction groups. Binary logistic regression analysis was performed to identify the outcome predictors after DA-CPR. A total of 2109 adult OHCA patients with DA-CPR were enrolled. Numbers of elderly patients in audio instruction and video instruction were 1260 (73.2%) and 214 (55.3%), respectively. Elderly patients and those outside the home or medical facility were more likely to receive video instruction. Favorable neurologic outcome was observed more in patients who received video-instructed DA-CPR (n = 75, 19.4%) than in patients who received audio-instructed DA-CPR (n = 117, 6.8%). The survival to discharge rate was also higher in video-instructed DA-CPR (n = 105, 27.1%) than in audio-instructed DA-CPR (n = 211, 12.3%). Video-instructed DA-CPR was significantly associated with neurologic recovery (aOR = 2.11, 95% CI 1.48–3.01) and survival to discharge (aOR = 1.81, 95% CI 1.33–2.46) compared to audio-instructed DA-CPR in adult OHCA patients after adjusting for age, gender, underlying diseases and CPR location. Video-instructed DA-CPR was associated with favorable outcomes in adult patients with OHCA in a metropolitan city equipped with sufficient experience and facilities**.**

## Introduction

Out-of-hospital cardiac arrest (OHCA) is one of the leading causes of death and millions of people each year die from OHCA. In Korea, the number of out-of-hospital cardiac arrests is increasing every year with increases in population age and cardiovascular diseases^1^. The incidence rate of OHCA was 37.5 per 100,000 population in 2006 and increased to 46.8 per 100,000 population in 2010. Since cardiac arrest patients can be resuscitated within four minutes after proper treatment without neurologic complications, bystander cardiopulmonary resuscitation (CPR) is a key factor in improving the survival and neurologic recovery of patients with OHCA^2^. Dispatcher-assisted CPR (DA-CPR) has been performed to improve the quality of bystander CPR. Song et al.^3^ reported that DA-CPR increased neurologic recovery rates (2.0% in 2010 and 3.6% in 2011) and discharge survival rates (7.1% in 2010 and 9.4% in 2011). In most cases of OHCA, DA-CPR has been carried out by audio instruction via a telephone call^4^. Due to the nature of audio instruction, the patients’ condition can only be identified through the caller’s eye. Accordingly, concerns are raised during audio-instructed DA-CPR such as whether (1) it is a cardiac arrest situation, (2) CPR is being performed correctly, and (3) the patient’s condition changed during CPR. In addition, the need for real-time feedback has increased in terms of the correct CPR position and rate. As mobile phone technology has developed, the number of video calls has increased in real life^5^. Therefore, there has been an issue of whether audio or video instruction is more effective for DA-CPR in real life. Theoretically, video-instructed CPR allows for real-time feedback between the dispatcher and caller and better identification of the patient’s condition than audio-instruction^6^. A meta-analysis conducted by Lin et al.^7^ revealed that video-instructed DA-CPR provided significantly improved chest compression rates with more correct hand positions. Recently, Lee et al.^5^ reported early clinical experience with video-instructed DA-CPR and analyzed the survival outcomes compared to those from audio-instructed CPR in Seoul, a metropolitan city in Korea, between January and December in 2017 In their study, video-instructed DA-CPR was associated with a higher rate of survival to discharge and favorable neurologic outcomes via unadjusted analysis. However, this relationship was not statistically significant in the adjusted analysis. We postulated that the inclusion of 3-month trial period results and incorrect CPR posture may have affected the outcomes despite the instruction provided. Therefore, further study is required to evaluate the clinical efficacy of video-instructed DA-CPR under circumstances of ample experience and facilities. In this study, we aimed to investigate whether video-instructed DA-CPR resulted in improved neurologic recovery and survival to discharge compared to audio-instructed DA-CPR in a city setting.

## Results

### Clinical characteristics of the patients

**Figure 1:**
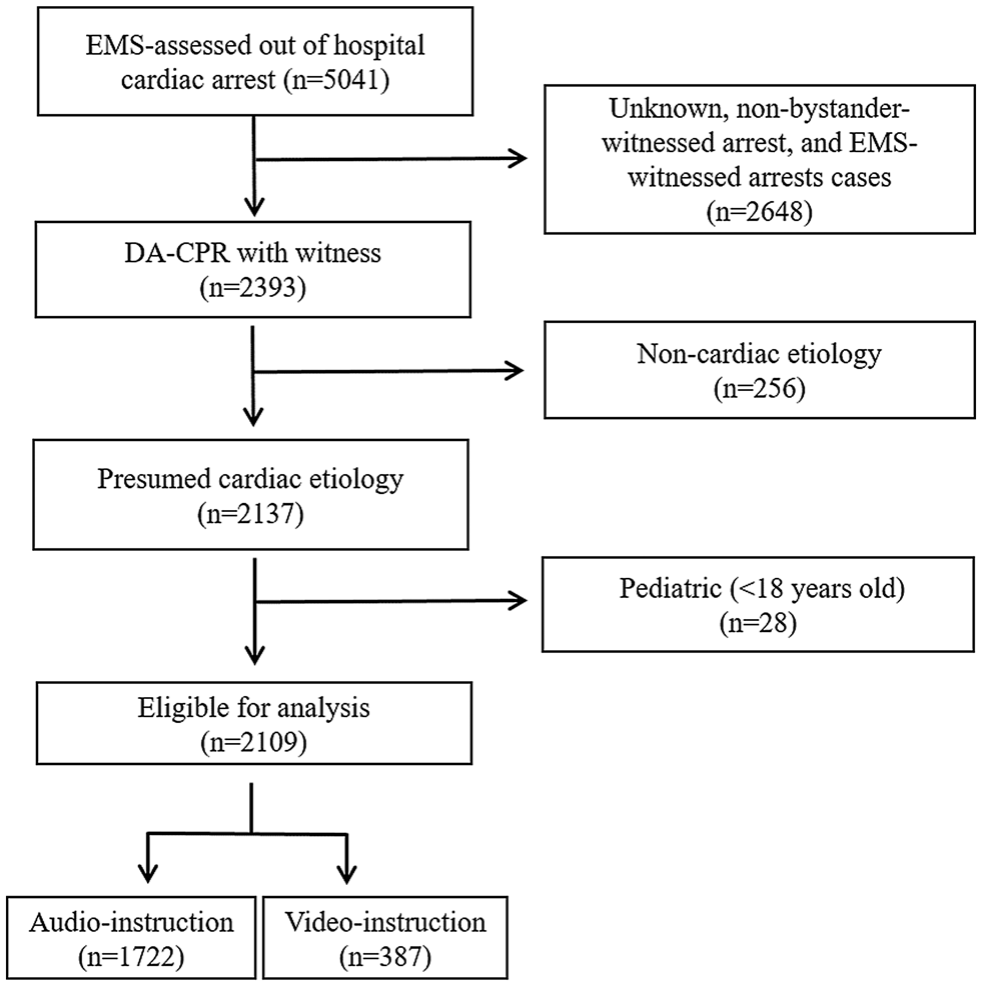
Flow diagram of the study population. EMS; emergency mdical service; DA-CPR, dispatcher-assisted cardiopulmonary resuscitation.

**Figure 2:**
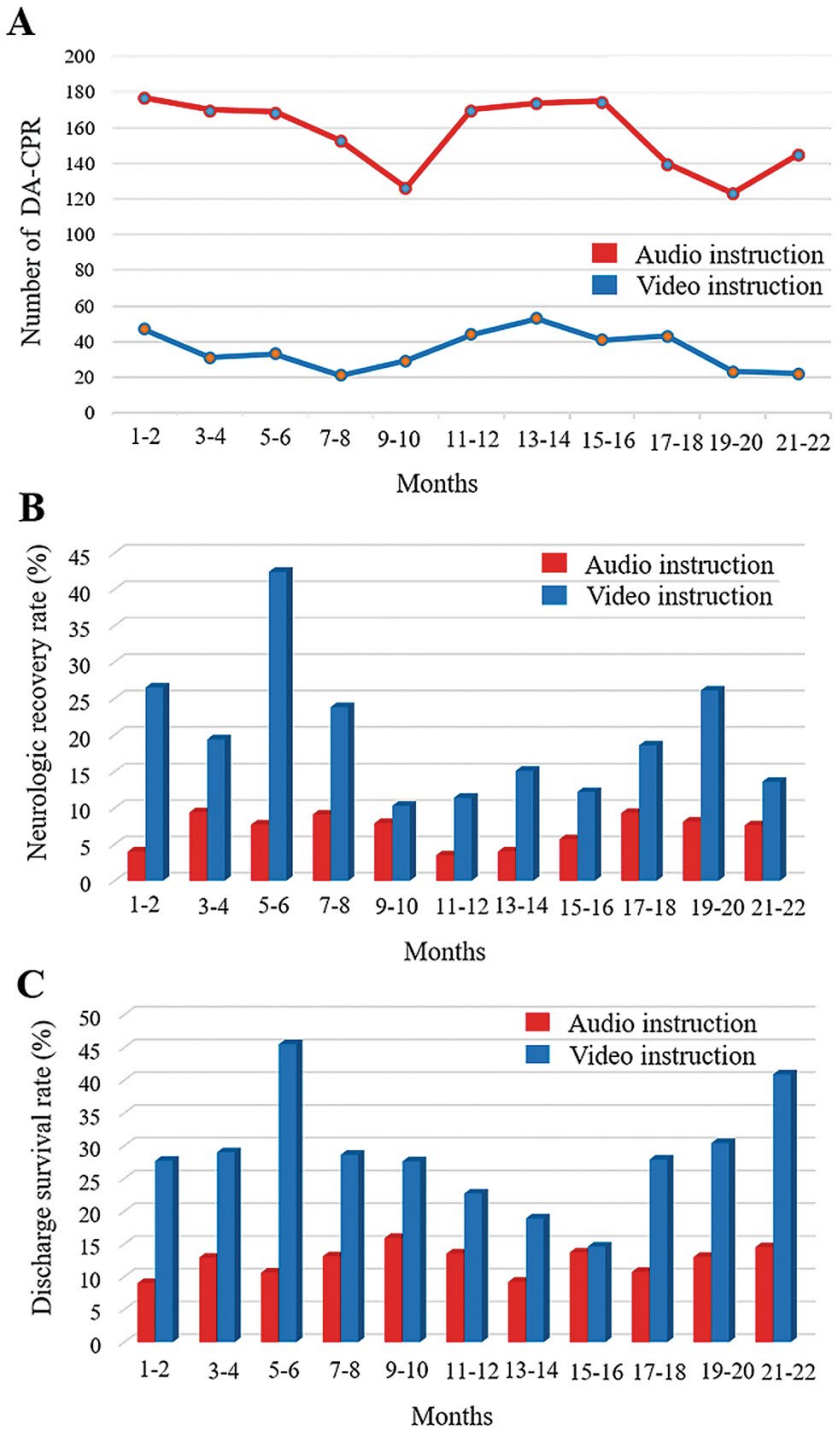
Trends in the bimonthly incidence of DA-CPR for adult patients with OHCA (**A**). Rates of favorable neurologic outcome (**B**) and survival to discharge (**C**) according to the CPR instructions delivered by video and audio-instructed DA CPR.

A total of 5041 cases of OHCA assessed by EMS were collected after screening the initial data from the Seoul Emergency Operation Center. After excluding unknown, non-bystander-witnessed arrest cases, and EMS-witnessed arrests cases (n = 2648), presumed non-cardiac etiology (n = 256), and pediatric cases (n = 28), 2109 cases were eligible for the final analysis (Fig. 1). Among them, 387 patients (18.3%) received video-instructed DA-PCR. The trends in the bimonthly incidence of DA-CPR are shown in Fig. 2. The number of video-instructed DA-CPR procedures ranged from 22 and 53 every 2 months, which was lower than that of audio-instructed DA-CPR between 123 and 177 (Fig. 2A).

**Figure 3:**
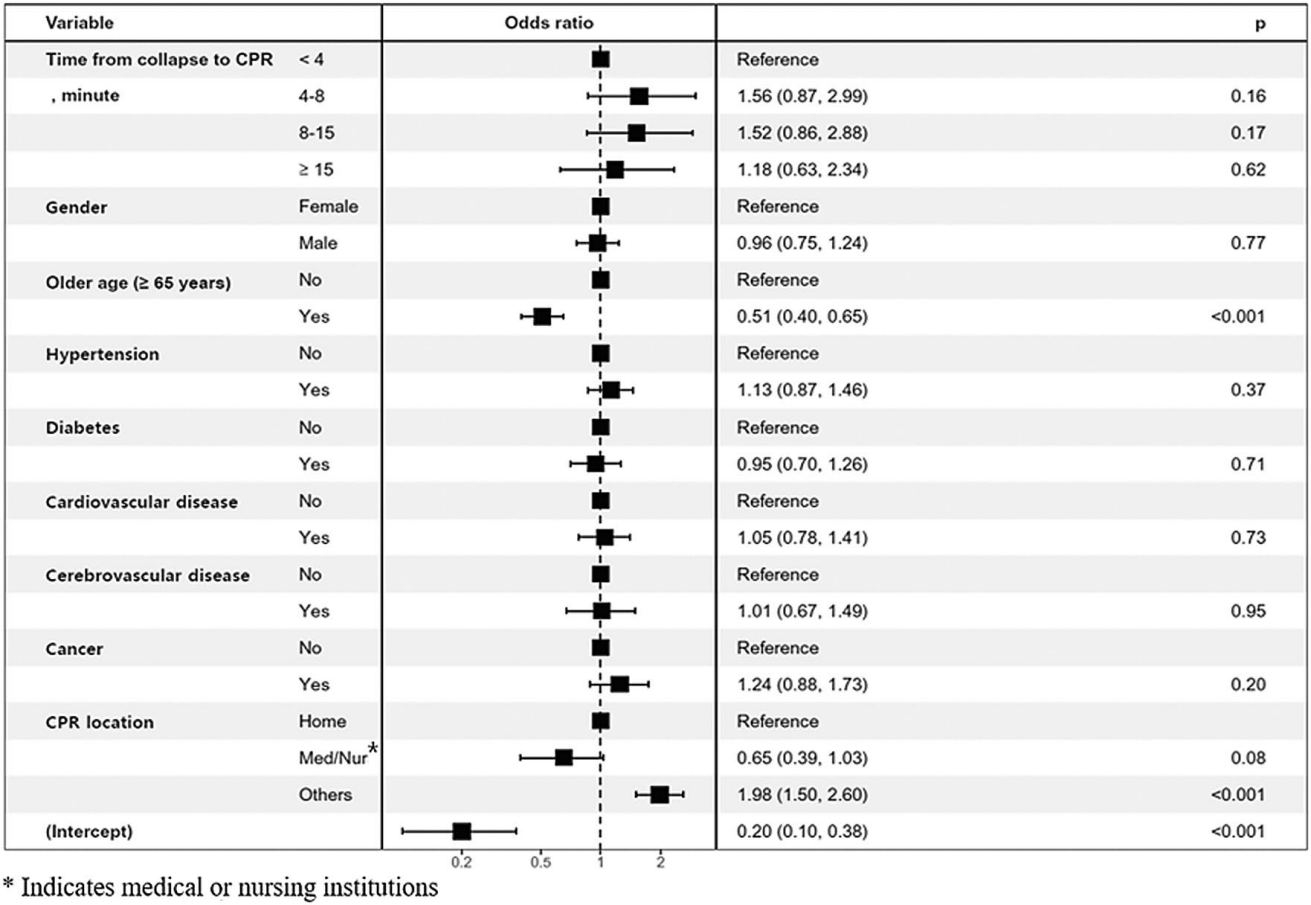
Factors influencing video-instructed DA-CPR in adult patients with OHCA.

The patients who received audio-instructed DA-CPR were older than those who received video-instructed DA-CPR (p < 0.0001). Elderly patients (> 65 years) and those outside the home or medical facility were more likely to be associated with the use of video-instructed DA-CPR (p < 0.0001) (Fig. 3).

### Outcome comparisons between video and audio-instructed DA-CPR

The rate of favorable neurologic outcome was 19.4% (75 out of 387) in patients who received video-instructed DA-CPR and 6.8% (117 out of 1722) in those who received audio-instructed DA-CPR (Table 1). Throughout all periods, video-instructed DA-CPR resulted in more than 2.4% to 34.7% higher rates of neurologic recovery compared to audio-instructed DA-CPR (Fig. 2B). Favorable neurologic outcome was associated with male gender (aOR = 1.92, 95% CI 1.26–2.92), coronary artery disease (aOR = 1.85, 95% CI 1.23–2.78), other CPR locations (neither home nor medical or nursing institution) (aOR = 2.33, 95% CI 1.63–3.32), and video instruction (aOR = 2.11, 95% CI 1.48–3.01) (Table 2).

**Table 1:** Clinical characteristics of adult patients with OHCA according to the instruction method.

Variables	Audio instruction (n = 1722)	Video instruction (n = 387)	p-value
**Clinical findings**			
Female	635 (36.9%)	124 (32.0%)	0.0734
Average age (mean ± SD), years	72.2 ± 14.7	64.9 ± 16.2	
Elderly (≥ 65 years)	1260 (73.2%)	214 (55.3%)	< 0.0001
Hypertension	616 (35.8%)	127 (32.8%)	0.2715
Diabetes mellitus	419 (24.3%)	81 (20.9%)	0.1551
Coronary artery disease	328 (19.0%)	71 (18.3%)	0.7503
Cerebrovascular disease	184 (10.7%)	33 (8.5%)	0.2068
Cancer	245 (14.2%)	54 (14.0%)	0.8889
**CPR location**			< 0.0001
Home	1239 (72.0%)	240 (62.0%)	
Medical/nursing institution	184 (10.7%)	21 (5.4%)	
Other places	299 (17.4%)	126 (32.6%)	
**Time from collapse to CPR**			0.0640
< 4 min	80 (4.6%)	14 (3.6%)	
≥ 4 to < 8 min	473 (27.5%)	122 (31.5%)	
≥ 8 to < 15 min	845 (49.1%)	197 (50.9%)	
≥ 15 min	324 (18.8%)	54 (14.0%)	
**First rhythm of paramedics**			< 0.0001
Asystole	869 (50.5%)	140 (36.2%)	
PEA	494 (28.7%)	94 (24.3%)	
VT/VF	359 (20.8%)	153 (39.5%)	
**Outcome**			
Out-of-hospital ROSC	239 (13.9%)	115 (29.7%)	< 0.0001
Survival to discharge	211 (12.3%)	105 (27.1%)	< 0.0001
Favorable neurologic outcome	117 (6.8%)	75 (19.4%)	< 0.0001

PEA, pulseless electrical activity; ROSC, return of spontaneous circulation; SD, standard deviation; VF, ventricular fibrillation; VT, ventricular tachycardia.

Data are presented as the numbers of subjects (percentage) for discrete and categorical variables and the mean ± standard deviation.

**Table 2:** Multivariable logistic regression analysis of the adjusted odds ratio of the favorable neurologic outcome in adult patients with OHCA.

Variable	Univariable model		Multivariable model	
	OR (95% CI)	p-value	aOR (95% CI)	p-value
Older age (yes vs. no)	0.13 (0.10–0.19	< 0.001	0.19 (0.13–0.27)	< 0.001
Gender (male vs. female)	3.05 (2.07–4.51)	< 0.001	1.92 (1.26–2.92)	< 0.003
**Time from collapse to CPR**				
< 4 min	1.0 (reference)	–	1.0 (reference)	–
≥ 4 to < 8 min	1.27 (0.65–2.49)	0.477	1.10 (0.53–2.31)	0.792
≥ 8 to < 15 min	0.66 (0.34–1.29)	0.225	0.63 (0.30–1.31)	0.213
≥ 15 min	0.23 (0.10–0.54)	< 0.001	0.26 (0.10–0.67)	0.005
**Underlying disease**				
Hypertension	0.72 (0.52–0.99)	0.046	1.36 (0.92–2.01)	0.123
Diabetes mellitus	0.45 (0.29–0.70)	< 0.001	0.57 (0.35–0.93)	0.025
Coronary artery disease	1.35 (0.95–1.92)	0.095	1.85 (1.23–2.78)	0.003
Cerebrovascular disease	0.36 (0.17–0.73)	0.005	0.65 (0.31–1.39)	0.271
Cancer	0.24 (0.12–0.50)	< 0.001	0.41 (0.19–0.87)	0.020
**CPR location**				
Home	1.0 (reference)	–	1.0 (reference)	–
Medical/nursing institution	0.39 (0.15–0.96)	0.041	0.52 (0.20–1.32)	0.169
Others	4.56 (3.34–6.23)	< 0.001	2.33 (1.63–3.32)	< 0.001
**Type of DA-CPR**				
Audio instruction	1.0 (reference)	–	1.0 (reference)	–
Video instruction	3.30 (2.41–4.52)	< 0.001	2.11 (1.48–3.01)	< 0.001

OR, odd ratio; aOR, adjusted OR; CI, confidence interval.

The rates of survival to discharge were 27.1% (105 out of 387) in video-instructed DA-CPR and 12.3% (211 out of 1722) in audio-instructed DA-CPR. Higher rates of survival to discharge was closely related to video instruction (aOR = 1.81, 95% CI 1.33–2.46) (Table 3). Based on the above results, video-instructed DA-CPR was significantly associated with favorable neurologic outcome and survival to discharge compared to audio-instructed CPR.

**Table 3:** Multivariable logistic regression of the adjusted odds ratio of the survival to discharge in adult patients with OHCA.

Variable	Univariable model		Multivariable model	
	OR (95% CI)	p-value	aOR (95% CI)	p-value
Older age (yes vs. no)	0.17 (0.13–0.22)	< 0.001	0.22 (0.17–0.30)	< 0.001
Gender (male vs. female)	2.55 (1.90–3.41)	< 0.001	1.77 (1.28–2.44)	< 0.003
**Time from collapse to CPR**				
< 4 min	1.0 (reference)	–	1.0 (reference)	–
≥ 4 to < 8 min	1.38 (0.79–2.41)	0.258	1.31 (0.71–2.43)	0.390
≥ 8 to < 15 min	0.69 (0.39–1.19)	0.183	0.70 (0.38–1.29)	0.255
≥ 15 min	0.30 (0.15–0.58)	< 0.001	0.34 (0.17–0.72)	0.005
**Underlying disease**				
Hypertension	0.65 (0.50–0.84)	0.001	1.10 (0.80–1.51)	0.568
Diabetes mellitus	0.47 (0.34–0.66)	< 0.001	0.60 (0.41–0.88)	0.009
Coronary artery disease	1.41 (1.06–1.88)	0.018	1.97 (1.41–2.75)	< 0.001
Cerebrovascular disease	0.42 (0.25–0.71)	0.001	0.69 (0.39–1.20)	0.189
Cancer	0.21 (0.11–0.37)	< 0.001	0.31 (0.17–0.58)	< 0.001
**CPR location**				
Home	1.0 (reference)	–	1.0 (reference)	–
Medical/nursing institution	0.90 (0.54–1.48)	0.675	1.15 (0.67–1.98)	0.603
Others	4.60 (3.54–5.98)	< 0.001	2.50 (1.85–3.38)	< 0.001
**Type of DA-CPR**				
Audio instruction	1.0 (reference)	–	1.0 (reference)	–
Video instruction	2.67 (2.04–3.48)	< 0.001	1.81 (1.33–2.46)	< 0.001

OR, odd ratio; aOR, adjusted OR; CI, confidence interval.

Additional results of the stratified analysis showed that video instruction was closely associated with favorable neurologic outcome of OCHA patients with unshockable rhythm (OR = 3.472; 95 CI 1.353–8.912). However, the effect was not prominent in achieving improved survival to discharge compared with audio instruction (OR = 1.620, 95% CI 0.843–3.116). For those with shockable rhythm such as VT and VF, video instructed DA-CPR was associated with favorable neurologic outcome (OR = 1.935; 95 CI 1.308–2.863) and survival to discharge (OR = 1.782; 95 CI 1.212–2.619).

## Discussion

At the scene, the rapid recognition of cardiac arrest and subsequent CPR delivered in the right position were important in achieving a favorable prognosis in patients with OCHA^8,9^. In cases of impending cardiac arrest, witnesses often misinterpret patient movements as signs of life when they encounter agonal breathing and tremors^10^. Accordingly, improvement in the initial CPR quality administered by a witness without delay at the scene is the main issue affecting the prognosis of OHCA patients. Theoretically, a video call can be advantageous for providing information and a better understanding of the dispatchers compared to audio calls. Johnsen et al.^6^ reported that video-assisted DA-CPR allowed for appropriate feedback to the dispatchers and improved rescuer compliance in a simulation study. A meta-analysis^7^ also revealed that video instruction allowed for a faster chest compression rate (104.8 in video instruction vs 80.6 in audio instruction) and a lower risk of incorrect hand positioning. However, the delayed initiation of chest compression was observed in video-instructed DA-CPR compared to audio-instructed DA-CPR. In the real clinical field, there has been a concern about the delay of CPR initiation during the video call connection process due to the adjustment of the camera angle, change of the screen, and monitoring the patient’s condition. To avoid delaying the CPR procedures at our institution, the first cycle of CPR was performed by audio instruction and then another caller was asked to switch to a video call. Although Lee et al.^5^ did not find significant differences in the time intervals between video and audio instruction (p = 0.12), further randomized controlled trials are necessary to identify the optimal protocol for video-instructed DA-CPR to obtain favorable neurological recovery and survival to discharge rates without a delay in initiating CPR.

The positive effects of video-instructed DA-CPR were demonstrated in a simulation study. However, the first real-world clinical study failed to prove the clinical efficacy of video-instructed DA-CPR on survival outcome^5^. In the analysis conducted after adjusting for all covariates, video-instructed DA-CPR did not increase favorable neurologic outcomes (OR = 1.28; 95% CI 0.73–2.26) and survival to discharge rates (OR = 1.20; 95% CI 0.74–1.94)^5^. However, that study investigated data from 2017, including the 3-month trial period results (Supplemental Figure S1). During the trial period between January to March in 2017, the favorable CPR outcome rates were lower in video-instructed DA-CPR than audio-instructed DA-CPR. When an emergency dispatcher gave CPR instructions using video calls during the trial period, the quality and characteristics of bystander CPR were different and inappropriate despite instructions outlined in the audio-instructed CPR protocol. To overcome these limitations, we transmitted the correct CPR position to the reporter’s video screen during video-instructed DA-CPR (Supplemental Figure S2). Therefore, more patients with correct CPR positions may have been included in this study than in the previous study^5^ and this affected the favorable neurologic outcome and survival to discharge rates.

Accumulated experience with video-instructed DA-CPR guidance can help enhance the effectiveness of the audio-instructed DA-CPR protocol. Video calls might not be possible depending on the caller considering the following conditions: (1) when caller was not good at using a mobile phone and could not be connected to a video call; (2) when the caller was confused and could not respond properly to the dispatcher’s instructions; (3) when there was no other person in the vicinity other than one caller; and (4) when caller was connected to a video call, it was difficult to transmit information on the site because proper operation such as camera switching was not possible. In this case, feedback data obtained through video-instructed DA-CPR can be used to help the witness perform appropriate resuscitation during audio-instructed DA-CPR. In addition, the dispatcher provides proper management due to real-time feedback via the video-call until the EMS arrives at the scene. Professional first-aid treatment such as airway maintenance and the infusion of fluid or vasopressors can also be performed. Accordingly, a more effective linkage between prehospital and hospital treatment is expected through video calls.

There might be concerns about how long the trial peiord will take for a video-instructed CPR system to perform properly in acutal clinical practice at places other than Seoul. Two big obstacles to implementing a video-instructed CPR were citizens’ perception and the presence of smartphone capable of making video calls. At the beginning, there was a tendency to reject the change from audio to video call instruction. However, these cases were gradually reduced by increasing public awareness through national news to promote video-instructed DA-CPR. Smartphone penetration rate in the population of Korea was approximatley 95%. Accordingly, it was relatively easy to change from audio to video call at the scence. If these two issues are resolved well, 3 months would be sufficient for the trial period. Additionally, it is necessary to solve potential problem in running the system during the same trial period.

There is a question about how much video instruction improves the CPR quality in actual emergency. We wanted to investigate this too. However, it was impossible to record in detail how CPR behavior changed by video instruction in a busy emergent situation. Thus, this study had a limitation in that it could not present numerically whether or not the specific CPR quality was increased by video instruction in terms of proper CPR position, compression depths and rates, and compressor postures in the real world. However, this was investigated through a simulation study focusing on the corrected effect by video instruction defined as the protocol with audio call-to-video call transition^11^. Lee et al.^11^ have reported significantly deeper depth of mean chest compression and improvement in adequate position (approximately 92% in the video instruction vs 82% in the audio instruction) with video-instructed DA-CPR compared to those with conventional audio-instructed DA-CPR in a simulation results of 131 volunteers. Especially, the effect was prominent for those taught by video instruction with a rapid transition from audio call. Nevertheless, it cannot accurately reflect actual clinical fields. Thus, further studies are needed to specifically investigate how much CPR quality is improved by video instructed DA-CPR in a real emergent situation.

There are some limitations to this study. First, this was a retrospective cohort study, although the number of CPR cases with video-instructed DA-CPR was the most up to date. Accordingly, the potential risk of selection bias for video-instructed DA-CPR could affect the CPR outcomes, particularly CPR location and results such as the first ECG rhythm recorded by EMS that arrived on site first. In our study, video-instructed DA-CPR was less performed at home and in medical or nursing institution. Thus, manpower might be insufficient to make a video call at home and the need to connect a video call might be less due to CPR knowledge of the staff members in medical and nursing institutions. The first documented ECG rhythm was closely related to CPR outcome. Hara et al.^12^ reported that favorable survival rate at 1 month was 21.3% in patients with pVT/VF, 2.7% in those with PEA, and 0.6% in those with asystole. Accordingly, video-instructed DA CPR could be a confounder to influence results due to increased shockable rhythm such as VT and VF. To address this possibility, we performed a stratified analysis (Supplemental Table S1). Results showed that video instruction was significantly associated with a favorable neurologic outcome (OR = 1.935; 95 CI 1.308–2.863) and survival to discharge (OR = 1.782; 95% CI 1.212–2.619) in OCHA patients with shockable rhythm. However, for those with asystole or PEA, the effect of video-instruction was not prominent in achieving improved survival to discharge compared to the effect of audio instruction (OR = 1.620, 95% CI 0.843–3.116). Second, CPR outcomes also can be affected by the degree of underlying medical diseases and fatigue of a bystander who is a CPR practitioner. The presence of coronary artery disease was associated with favorable outcomes after CPR in our study. The family of patients with coronary artery disease may be more likely to have appropriate CPR knowledge or less fear of performing CPR than those without family members with coronary artery disease. When CPR is taught by audio-instruction, the dispatcher cannot actually check the fatigue of a bystander who is a CPR practitioner. Thus, it is impossible to actively designate someone to change CPR regardless of the number of on-site personnel. In the case of video calls, the dispatcher was able to visually check bystander fatigue. In addition, CPR could be shifted by actively pointing out people around by the dispatcher. Therefore, bystander fatigue could be a confounder to the interpretation of results. However, it is difficult to accurately measure the degree of fatigue in a real emergency situation, although fatigue worsens over time. Thus, the effect of fatigue on CPR outcome according to the instruction method was indirectly analyzed using a stratified analysis between the two groups (initial collapse to CPR time < 8 min vs. ≥ 8 min), although the initial collapse to CPR time and bystander CPR time did not exactly match  (Supplemental Table S2). Interestingly, video instruction was significantly associated with favorable neurologic outcome (OR = 2.088, 95% CI 1.212–3.597) and survival to discharge (OR = 2.174, 95% CI 1.292–3.658) in OCHA patients with initial collapse to CPR time ≥ 8 min. However, these associations were insignificant for those with initial collapse to CPR time < 8 min. These results suggest that CPR outcome between video- and audio-instructed DA-CPR could be different according to fatigue. Additionally, the time from collapse to CPR initiation relied on the EMS interview with witnessed bystander CPR. Although there was no other way to realistically estimate the time interval which was checked in twice in our study, the exact time for a cardiac arrest and the exact time for initiating the CPR could be estimated incorrectly^5^. Therefore, randomized controlled studies that control factors affecting outcomes as possible confounding factors are needed in the future to accurately correlate video-instructed DA-CPR with outcomes.

## Conclusion

Video-instructed DA-CPR was associated with favorable outcomes in adult patients with OHCA in a metropolitan city equipped with sufficient experience and facilities. Randomized controlled trials are necessary to confirm our results.

## Methods

### Emergency system in Seoul

Seoul is a large city with a population of approximately 10 million people within a total of 605 km^2^. The number of 119 reports, the code indicating an emergency, per year is approximately 2 million, of which approximately 600,000 were disaster reports^5^. To establish an effective disaster prevention system, the Seoul Emergency Operations Center (SEOC) was established in 2002. The SEOC is in charge of disasters such as typhoons, floods, heavy rain, strong wind, heavy snow, and earthquakes as well as emergency medical consultation and medical guidance in situations that threaten the safety and property of Seoul citizens. Currently, the system has been established with 24 fire departments and 151 emergency medical services. After the standard DA-CPR protocol is applied^5^, a more systematic and immediate consultation is performed by professional medical staff. The emergency command system is operated by following a written protocol. First, when a call comes in, the primary situation call-taker receives the report and roughly ascertains the main contents of the report. Then, the call-taker confirms the chief complaint and delivers the dispatch order to each EMS. In particular, if emergency treatment is needed, including instruction after a prehospital cardiac arrest, a phone call to an emergency medical dispatcher is made immediately. The dispatcher ascertains the patient's condition and provides first aid guidance as necessary. In an out-of-hospital cardiac arrest, the secondary dispatcher immediately executes a dispatcher CPR order and shares information about the patient's condition with the emergency unit until they arrive at the scene. When the paramedics arrive, they contact to the first aid doctor in the center by phone, providing information on the patient’s condition to the doctor and performing procedures according to the medical guidance provided (Supplemental Figure S3). Informed consent for study participation was obtained.

### Video-instructed DA-CPR

This is a real-time support system of “Smart Video First Aid Instruction” where a 119 consultant provides first aid instructions through a video call with a caller until ambulance arrives. The system consists of three steps as follows: (1) after providing CPR instruction to the caller, the 119 consultant identifies the patient’s condition through a video call; (2) the 119 consultant provides proper CPR techniques including the rescuer’s position, hand position, and the adequate chest compression rate and depth until the emergency medical service (EMS) arrives; and (3) an emergency medicine doctor affiliated with the 119 situation room in SEOC constantly interacts with the EMS from the scene to the hospital. Video-instructed DA-CPR was attempted if the following conditions were met: (1) the presence of two or more bystanders and (2) the availability of a video call when CPR guidance based on the telephone consultation protocol was initiated by the secondary dispatcher^5^. In such case, thee callers were reconnected by a video call. The system was conducted on a trial basis from January 16 to March 31, 2017. During the trial period, the system did not significantly increase the rates of favorable neurologic outcome and survival to discharge (Supplemental Figure S1). However, there were positive effects between the dispatcher and caller via video-instructed DA-CPR, such as real-time feedback, better identification of the patient’s condition, and better guidance when the caller was hesitant or panicked in the emergency situation. Therefore, an official decision was made to implement the system by extending the period.

### Data collection, variables, and study outcomes

We collected data from the EMS-assessed out-of-hospital cardiac arrest database in the SEOC between January 1, 2018 and October 31, 2019 (Fig. 2). Inclusion criteria were as follows: (1) witnessed CPR; (2) presumed cardiac origin; (3) adult patients more than 18 years old; (4) DA-CPR instructed by dispatcher. Exclusion criteria were as follows: (1) non-witnessed cases; (2) non-cardiac etiologies; and (3) cases without an attempt for resuscitation^5^. Cardiac arrest was divided into two groups of presumed cardiac origin and non-cardiac origin. Non-cardiac origin was defined as a cardiac arrest due to external causes, respiratory diseases, stroke, or malignant tumor based on hospital records^13^. Records regarding demographical factors, underlying diseases, CPR location, and time from collapse to CPR were reviewed. The demographics included gender and age. Age over 65 years was used to define elderly patients^14^. The underlying diseases were hypertension, diabetes mellitus, coronary artery disease, cerebrovascular disease, and cancer. The CPR location was divided into three groups of home, medical or nursing institutions, and other places. The time from collapse to CPR was categorized into four groups of time < 4 min, ≥ 4 and < 8 min, ≥ 8 and < 15 min, and > 15 min^15^.

The primary outcome was the difference in favorable neurologic outcome in the video-instructed DA-CPR and audio-instructed DA-CPR. The favorable neurologic outcome was defined as cerebral performance category (CPC) scores of 1 and 2 measured at hospital discharge, indicating independent activities of daily life after CPR^15^. The secondary outcome was the survival to discharge rates between the two instruction methods. We also compared the differences in the first rhythm recorded by the paramedics and the return of spontaneous circulation (ROSC) before arriving at the hospital. Study design was performed according to the principles of the Declaration of Helsinki and were approved by the Institutional Review Board of the Chuncheon Sacred Heart Hospital (IRB No. 2020-09-016). All methods were performed in accordance with the relevant guidelines and regulations in manuscript.

### Statistical analysis

Continuous variables are described as the mean and standard deviation (SD). A Chi-square or Student’s t-test was performed to find differences according to the type of CPR instruction. Univariate analysis was performed to find relevant factors for outcomes such as favorable neurologic outcome and survival to discharge in adult OHCA patients. Multivariable logistic regression analysis was performed further to confirm the statistical independence of variables after adjusting for age, gender, underlying diseases, time from collapse to CPR, and CPR location. The results were expressed as adjusted odds ratios (aORs) with 95% confidence intervals (CIs). In this study, the first rhythm itself could be a confounder influencing results of this study. To address this possibility, we further performed a stratified analysis using the Cochran-Mantel–Haenszel Method to examine the primary association of interest at different levels of a confounding factor (Supplemental Table S1). Statistics were performed with R (version 3.6.1) and MedCalc (http://www.Medcalc.org) with a statistical significance indicated at p < 0.05.

### Compliance with ethical standards

Data collection and study design were performed according to the principles of the Declaration of Helsinki and were approved by Coordinating Ethnics Committee of the Chuncheon Sacred Heart Hospital.

## Supplementary Information

Supplementary Information.

## Data Availability

Data are available from the corresponding author (SK) upon ethical approval from the IRB of the participating hospital.
