# Supplementary material for: The effect of video-instructed versus audio-instructed dispatcher-assisted cardiopulmonary resuscitation on patient outcomes following out of hospital cardiac arrest in Seoul
Source: Sci Rep. 2021 Jul 30;11:15555. doi: 10.1038/s41598-021-95077-5 (PMC8324920; doi:10.1038/s41598-021-95077-5)
Supplement: Supplementary file 1 — Supplementary Information. [file 41598_2021_95077_MOESM1_ESM.docx]

**Supplemental Data**

**Figure S1.** Monthly rates of favorable neurologic recovery (A) and survival to discharge (B) between video and audio-instructed DA-CPR in 2017. Video-instructed CPR, entitled “Smart Video First Aid Instruction,” was performed on a trial basis from January 16 to March 31 in 2017 in the Seoul Emergency operation center. After the trial period was over, video-instructed DA-CPR exhibited favorable neurological outcome rates 15.4% to 30.0% higher and survival to discharge rates 15.3% to 30.0% higher than audio-instructed DA-CPR except for in October.


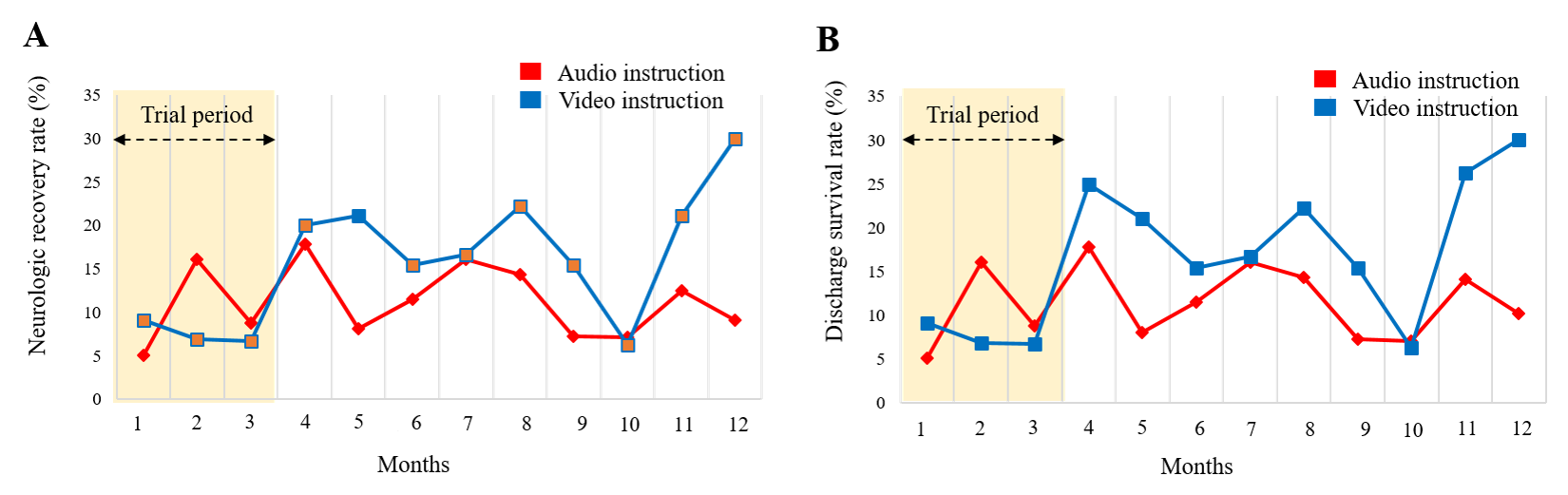


**Figure S2.** The correct CPR posture instructions such as the hand (A), chest (B), and compression postures (C) are transmitted to the reporter’s video screen during the video-instructed DA-CPR.


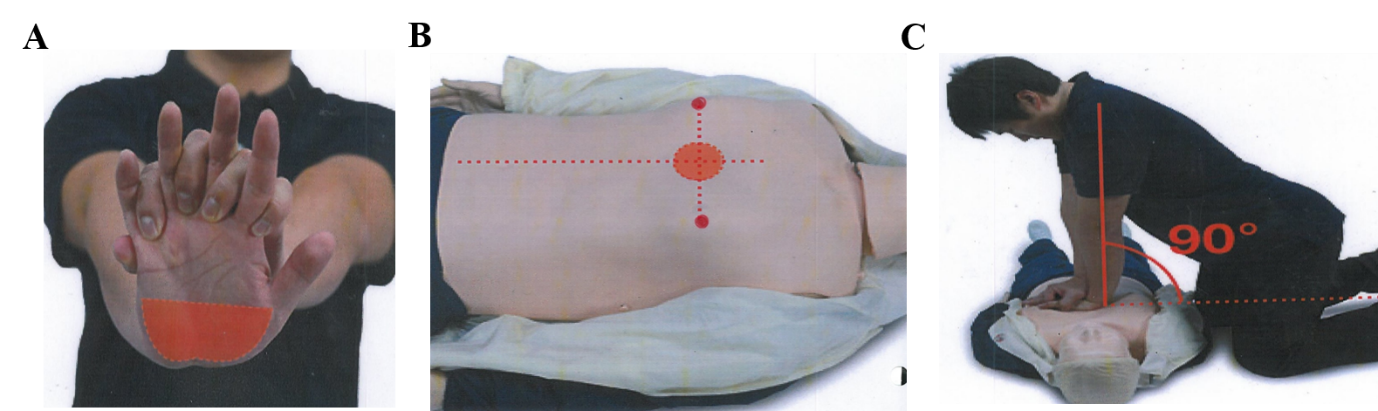


**Figure S3.** Illustration of cardiac arrest patient information protocol performed in the emergency system in Seoul. The identification of patient condition by an emergency medical dispatcher who is a 119 consultant. The medical dispatcher explains proper CPR techniques until EMS arrival via video or audio call, shown as video and audio-instructed DA-CPR. After EMS arrival, an emergency medicine doctor affiliated with the 119 situation room in the Seoul emergency operation center interacts with EMS to monitor the patient’s condition and provide professional first-aid treatment until hospital arrival.

**
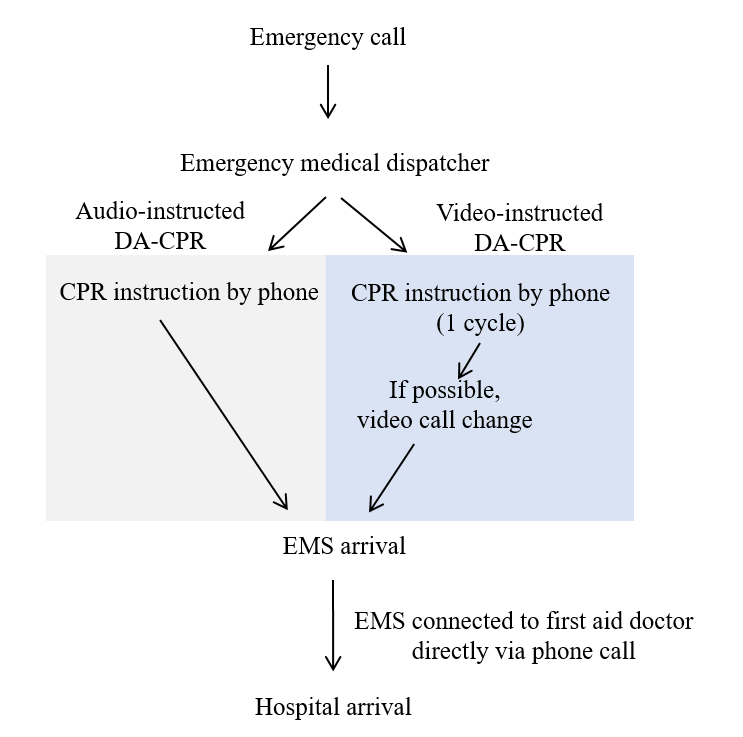
**

**Supplemental Table S1.** Stratification analysis to determine effects of instruction methods and shockable rhythm on outcomes of adult patients with OHCA.

| First rhythm |  | Video instruction (vs Audio) |
| --- | --- | --- |
| of paramedics | Outcomes | Odds ratio (95% Confidence interval) |
| Asystole or PEA | Favorable neurologic outcome | 3.472 (1.353-8.912) |
|  | Survival to discharge | 1.620 (0.843-3.116) |
| VT/VF | Favorable outcome | 1.935 (1.308-2.863) |
|  | Survival to discharge | 1.782 (1.212-2.619) |

PEA, pulseless electrical activity; VF, ventricular fibrillation; VT, ventricular tachycardia.

**Supplemental Table S2**. Stratification analysis to determine effects of instruction methods and initial collapse to CPR time on outcomes of adult patients with OHCA.

| Time from collapse |  | Video instruction (vs Audio) |
| --- | --- | --- |
| to CPR | Outcomes | Odds ratio (95% Confidence interval) |
| < 8 min | Favorable neurologic outcome | 1.647 (0.929-2.920) |
|  | Survival to discharge | 1.241 (0.691-2.230) |
| ≥ 8 min | Favorable outcome | 2.088 (1.212-3.597) |
|  | Survival to discharge | 2.174 (1.292-3.658) |

CPR, cardiopulmonary resuscitation.
